# Reactive Strength Index, Rate of Torque Development, and Performance in Well-Trained Weightlifters: A Pilot Study

**DOI:** 10.3390/jfmk8040161

**Published:** 2023-11-20

**Authors:** Giorgos Anastasiou, Marios Hadjicharalambous, Gerasimos Terzis, Nikolaos Zaras

**Affiliations:** 1 Human Performance Laboratory, Department of Life Sciences, School of Life and Health Sciences, University of Nicosia, Nicosia 2417, Cyprushadjicharalambous.m@unic.ac.cy (M.H.); 2 Sports Performance Laboratory, School of Physical Education and Sport Science, National and Kapodistrian University of Athens, 157 72 Athens, Greece; gterzis@phed.uoa.gr

**Keywords:** snatch, clean and jerk, contact time, power

## Abstract

The purpose of this study was to investigate the correlation between the reactive strength index (RSI) using the drop jump (DJ) and the isometric rate of torque development (RTD) with weightlifting performance in national-level weightlifters. Seven male weightlifters (age: 28.3 ± 5.7 years, body mass: 80.5 ± 6.7 kg, body height: 1.73 ± 0.07 m) participated in this study. Measurements were performed 2 weeks prior to the national championship and included the countermovement jump (CMJ), the squat jump (SJ), the DJ from three different drop heights (20, 30, and 40 cm), and the isometric peak torque (IPT) and RTD. Performance in CMJ and SJ was significantly correlated with weightlifting performance (r ranging from 0.756 to 0.892). Significant correlations were found between weightlifting performance with DJ contact time (r ranging from −0.759 to −0.899) and RSI (r ranging from 0.790 to 0.922). Moreover, the best RSI was significantly correlated with the snatch (r = 0.921, *p* = 0.003) and total performance (r = 0.832, *p* = 0.020). Small to very large correlations were found between IPT and RTD with weightlifting performance (r ranging from 0.254 to 0.796). These results suggest that RSI and contact time variables from DJ may predict weightlifting performance in well-trained weightlifters. Additionally, IPT and RTD may provide useful insights into the neuromuscular fitness condition of the weightlifter.

## 1. Introduction

Olympic weightlifting is a power-demanding sport consisting of two lifts: the snatch and the clean and jerk. During the snatch, the athlete is required to lift the barbell from the ground and overhead in one consecutive movement [1]. In addition, the clean and jerk consists of two different movements: the clean, which requires the athlete to lift the barbell from the ground to the shoulders, and the jerk, which requires the athlete to bring the barbell from the shoulders to overhead [2]. Both the snatch and clean and jerk lifts are highly technical multi-joint movements where the entire neuromuscular system of the weightlifter must be simultaneously activated for a successful lifting attempt [3]. 

In Olympic weightlifting training, athletes routinely utilize heavy loads (≥80% of 1 repetition maximum (RM)), with an intentionally fast movement of lifting velocity [4], to attempt to enhance performance during main competitions. This strategy is not only applied in the snatch and the clean and jerk but also in other weightlifting derivatives (back squat, front squat, pulls, etc.). This type of training may lead to a significant increase in power (i.e., vertical jumps) and in the rate of force development (RFD) [5,6,7]. Several studies, for example, have shown that vertical jumps such as the countermovement jump (CMJ), the deep squat CMJ, and the squat jump (SJ) may significantly predict performance in the snatch and clean and jerk. Indeed, a significant correlation was observed between weightlifting performance and CMJ variables [6,7,8,9,10,11], whereas deep CMJ and SJ may also be valid predictors of weightlifting performance [8,12,13]. Furthermore, a recent meta-analysis revealed that CMJ and SJ power production had a significant correlation magnitude to weightlifting performance, reaching nearly perfect values (r = 0.92) [14]. Although the link between CMJ and SJ with weightlifting performance has been extensively researched, the correlation between drop jump (DJ) capability and weightlifting performance remains largely unexplored. The DJ is considered a fast stretch–shortening cycle movement that produces a rapid eccentric phase and a faster transition to the concentric phase [15], which is similar to the weightlifting movements. A crucial variable derived from the DJ analysis is the reactive strength index (RSI), which can be calculated as the quotient of DJ height and ground contact time [16,17]. A previous study on male NCAA Division I basketball players showed that the RSI was correlated with the vertical stiffness of the lower musculoskeletal system during various DJ heights [17]. Although data are scarce for weightlifters, it could be hypothesized that the DJ may be a useful test to predict weightlifting performance, even though such a premise needs further investigation. 

Performance in weightlifting requires a high RFD in short time frames ranging from 0 to 250 milliseconds [18,19,20]. The RFD is a parameter that shows how quickly an athlete can apply his/her maximum force, and it is calculated by the force–time curve [7,21,22]. Significant correlations have been found between weightlifting performance with RFD measured through mid-thigh pull [9,13,21] and isometric leg press [7,10,11]. In addition, a recent meta-analysis showed that the RFD calculated from mid-thigh pulls may have a significant correlation magnitude to weightlifting performance, reaching large values (r ranging from 0.51 to 0.60) [14]. Although these tests are multi-joint and sports-specific, several laboratories are equipped with isokinetic machines that can measure angular velocities in single-joint movements with high accuracy [23]. Additionally, it is a common strategy to evaluate athletes’ preparedness before competitions through isokinetic measurements. However, data are scarce regarding the correlation between single-joint movements, like the isometric knee extension and weightlifting performance. A study of sixty-seven adolescent weightlifters showed significant correlations between weightlifting performance and isokinetic knee extension force at 60, 90, and 180 deg/sec (r = 0.597, 0.693, and 0.725, respectively) [23], but no correlation was presented between the rate of torque development (RTD) and weightlifting performance. Consequently, whether a correlation exists between the knee extension isometric peak torque (IPT) and the RTD with weightlifting performance remains unexplored.

The aim of the present study was to investigate the correlation (a) between RSI and weightlifting performance and (b) between knee extension IPT and RTD with weightlifting performance. It was hypothesized that the RSI and knee extension IPT and RTD might be valid predictors of weightlifting performance. 

## 2. Materials and Methods

### 2.1. Participants

Seven male weightlifters (N = 7, age: 28.3 ± 5.7 years, body mass: 80.5 ± 6.7 kg, body height: 1.72 ± 0.07 m, personal best in snatch: 105.6 ± 14.2 kg, personal best in clean and jerk: 131.5 ± 19.6 kg) with 4.6 ± 2.2 years of competitive experience participated in the study. Three of the athletes were members of the national weightlifting team and holders of the national records in their individual bodyweight categories. All athletes participated in national and international weightlifting competitions. Inclusion criteria were as follows: (a) absence of any cardiovascular, orthopedic, and neuromuscular issues; (b) systematic weightlifting training and regular participation in competitions; (c) absence of any illegal drug use. Athletes were fully informed of the risks and benefits of the study prior to entry, and they signed an institutionally approved informed consent. All procedures were in accordance with the 1975 Declaration of Helsinki as revised in 2000 and were approved by the national ethics committee of Cyprus (project number ΕΕΒΚ/ΕΠ/2020/55).

### 2.2. Design

This study focused on the investigation of correlations between the DJ, the knee extension IPT, and RTD with weightlifting performance. Seven well-trained male weightlifters visited the laboratory on two different occasions within a week. During the first visit, anthropometric characteristics and a familiarization session of the vertical jumps and the knee extension isometric test were performed. During the second visit, measurements of the CMJ, the SJ, the DJ, and the knee extension isometric test were performed. On a different day, all athletes performed the 1-RM test in the snatch and clean and jerk in their training facilities. A correlation analysis was performed to investigate the relationships between variables.

### 2.3. Olympic Weightlifting Performance

Performance in weightlifting (snatch and clean and jerk) was measured at the training facilities of the athletes during the afternoon hours at a standard temperature of ~24 °C [24]. Athletes performed the 1-RM test in the snatch and clean and jerk according to the international regulations of the International Weightlifting Federation. Specifically, the 1-RM test started with the snatch. After a self-selected warm-up with static and dynamic stretching exercises, athletes performed 3–4 sets of 5–6 repetitions with an empty barbell. Then, the athletes performed 2 sets of 5 repetitions with 50% of the predicted 1-RM and 1 set of 2–3 repetitions at 65%, 75%, and 85% of the predicted 1-RM. Then, single repetitions were performed at 90% and 95% of the predicted 1-RM. Three maximum attempts were given to athletes after 95% of their individual predicted 1RM for achieving their maximum effort. Fifteen minutes after the snatch, the 1-RM test in the clean and jerk was performed, similarly to 1-RM snatch test, as described above. During the 1-RM attempts, a certified weightlifting coach was present to provide feedback to the athletes. The best performance in the snatch and in the clean and jerk was used for the statistical analysis. Total performance was expressed as the sum of the snatch and the clean and jerk in kilograms.

### 2.4. Countermovement Jumps

Laboratory measurements began with the CMJs. Briefly, after 5 min warm-up on a stationary bicycle and following several dynamic stretching exercises, athletes performed 3 sub-maximal intensity CMJs. Following 3 min of rest, the athletes performed 4 maximal CMJs (Optojump Modular System, Warwickshire, UK) with 2 min rest between each attempt. More specifically, the athletes remained in a standing position with arms akimbo; from this position, the athletes performed an individual self-selected semi-squat and jumped as high as possible. Data were recorded and analyzed to calculate the maximum vertical jump height, the power output [Power = (51.9·CMJ height in cm) + (48.9·Body Mass in kg) − 2007], and the power per body mass [25]. The highest jump height was used in the statistical analysis. The intra-class correlation coefficients (ICC) for the CMJ height, the power production, and the power per body mass were 0.989 [95% Confident intervals (CI): Lower = 0.957, Upper = 0.997], 0.980 (95% CI: Lower = 0.985, Upper = 0.990), and 0.981 (95% CI: Lower = 0.978, Upper = 0.991), respectively.

### 2.5. Squat Jumps 

Following the CMJs, athletes performed the SJs. Similarly to the CMJs test, athletes performed 3 SJs attempts with sub-maximal intensity followed by 4 SJs attempts with maximal intensity. More specifically, the athletes remained in a standing position with arms akimbo; then, the athletes performed an individual self-selected semi-squat and remained motionless until the researcher gave them the instruction to jump. No countermovement was allowed. The highest jump height was used in the statistical analysis. Data were recorded and analyzed (Optojump Next, Warwickshire, UK) for calculating the maximum vertical jump height, the power output [Power = (60.7·SJ height in cm) + (45.3·Body Mass in kg) − 2055], and the power per body mass [25]. The ICC for the SJ height, the power production, and the power per body mass were 0.979 (95% CI: Lower = 0.962, Upper = 0.998), 0.985 (95% CI: Lower = 0.989, Upper = 0.991), and 0.989 (95% CI: Lower = 0.968, Upper = 0.995), respectively.

### 2.6. Drop Jumps 

Ten minutes after the SJ test, the athletes performed the DJ test. Three different drop heights were used: 20, 30, and 40 cm. All athletes were familiar with the technique of the DJ since they had performed it before, in previous similar measurements, in the same laboratory. For warming-up purposes, two DJ attempts with sub-maximal intensity were allowed from 20 cm drop height for all athletes, and after 3 min, the athletes performed 3 DJ attempts with maximal intensity with arms akimbo from all drop heights with a randomized order (Optojump Modular System, Warwickshire, UK). Two minutes of rest were allowed between each attempt. Athletes stepped on the box with arms akimbo and projected their limb of choice in front of them and outside the box. Then, they were instructed to let their body fall down with both their feet touching the ground simultaneously. Researchers also instructed the athletes to minimize the ground contact time as much as possible (floor is lava) and then to jump as high as possible. Data were recorded and analyzed to calculate the time flight, the contact time, the jump height, the RSI, and the reactive strength ratio (RSR) [26]. The DJ with the best RSI was used for the statistical analysis [27]. The ICCs for the time flight, the contact time, the jump height, the RSI, and the RSR were 0.987 (95% CI: Lower = 0.934, Upper = 0.998), 0.961 (95% CI: Lower = 0.795, Upper = 0.993), 0.987 (95% CI: Lower = 0.934, Upper = 0.998), 0.995 (95% CI: Lower = 0.971, Upper = 0.999), and 0.996 (95% CI: Lower = 0.980, Upper = 0.999), respectively. 

### 2.7. Isokinetic Knee Extension Peak Torque and Rate of Torque Development

Fifteen minutes after the DJs, athletes performed the isometric knee extension measurement on an isokinetic dynamometer (HUMAC NORM isokinetic extremity system, Massachusetts, USA) for the evaluation of the quadriceps maximum IPT and RTD. The athletes were seated on the isokinetic dynamometer chair, and straps were used to ensure the stable position of the shoulders, the hips, and the non-tested leg. The tested leg was determined during the familiarization session [28]. Additionally, both hips were at 110° flexion while the knee angle was set at 60° flexion (0° = full extension) [29,30]. Three submaximal effort trials were performed with progressively increasing force, and then 3 maximal effort trials were performed. Athletes were instructed to apply their maximum force as fast as possible and to sustain it for 3 s. Real-time visual feedback of the torque applied was provided for each effort via a computer monitor placed just in front of the athlete, while athletes received verbal encouragement to apply their maximum force. Data from the isometric measurement were recorded and analyzed from the isometric torque–time curve. Maximum IPT was calculated as the greater torque generated from the torque–time curve, while RTD was calculated as the mean tangential slope of the torque–time curve in specific windows of 0–20, 0–40, 0–60, 0–80, 0–100, 0–120, 0–150, 0–200, and 0–250 milliseconds. The ICC for the IPT was 0.990 (95% CI: Lower = 0.964, Upper = 0.998), and for the RTD, it was 0.893 (95% CI: Lower = 0.649, Upper = 0.972).

### 2.8. Statistical Analysis

All data are presented as means ± SD. Performance in the snatch, the clean and jerk, and the total were collected as absolute values and transformed according to the Sinclair formula, which is a polynomial equation for weightlifters and is used as a method of obviating body mass differences in weightlifting total [13,31]. Pearson’s r product-moment correlation coefficient was used to explore the relationships between the weightlifting performance (Sinclair values) with the CMJ, the SQJ, the DJ, the IPT, and the RTD. In addition, Hopkins scales were used to investigate the magnitude of effect for the correlations: trivial < 0.10; small < 0.10–0.29; moderate ≤ 0.30–0.49; large ≤ 0.50–0.69; very large ≤ 0.70–0.89; and nearly perfect ≥ 0.9 [32]. A three-way analysis of variance for repeated measures was used to examine differences between the DJ heights (20, 30 40 cm) with a Bonferroni correction. Due to the small sample size, the Hedges g effect size was calculated. Reliability for all measurements was performed using a two-way random effect ICC with 95% CI. Significance was accepted at *p* ≤ 0.05. 

## 3. Results

All weightlifters completed all performance tests without experiencing any injury. Table 1 presents the results from the snatch, the clean and jerk, and the total expressed both with absolute and Sinclair values, as well as the variables calculated from the CMJ, the SJ, the IPT, and the RTD. 

**Figure 1:**
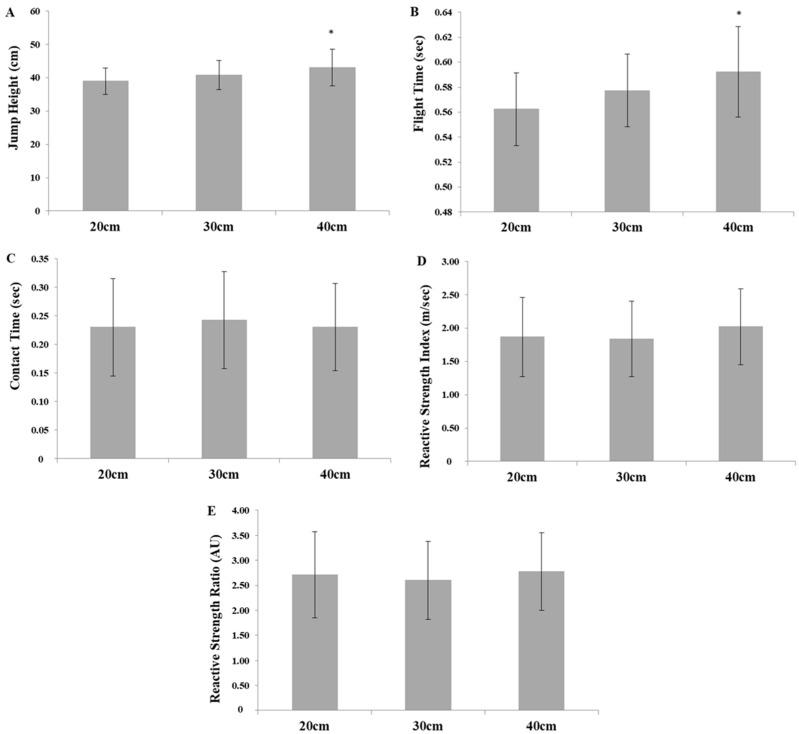
Results from the drop jump test: (**A**) jump height, (**B**) flight time, (**C**) contact time, (**D**) reactive strength index, and (**E**) reactive strength ratio; * *p* < 0.05, significant difference between 40 and 20 cm drop heights.

Results from the DJ are presented in Figure 1. Specifically, the flight time was significantly longer for the 40 cm condition compared with 20 cm (*p* = 0.013, *g* = 0.888), but not when compared with the 30 cm drop height (*p* = 0.065, *g* = 0.436). No significant difference was found for contact time between conditions (*p* = 0.058). Jump height was significantly greater for the 40 cm compared with the 20 cm condition (*p* = 0.015, *g* = 0.848), but not when compared with the 30 cm drop height (*p* = 0.072, *g* = 0.428). No significant difference was found for the RSI (*p* = 0.128) and the RSR (*p* = 0.102) between all conditions. Almost all athletes achieved their best RSI and RSR from the 40 cm drop height, except for one who achieved his best RSI and RSR from the 20 cm drop height. Consequently, the optimum average drop height for the best RSI for all athletes was 37.1 ± 7.6 cm. 

**Figure 2:**
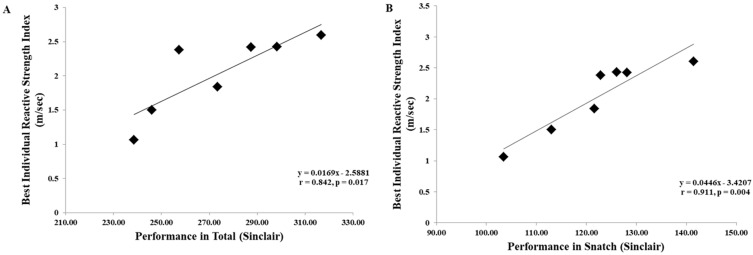
Correlation between the best individual reactive strength index with (**A**) total and (**B**) snatch.

All variables from the CMJ and the SJ were significantly positively correlated with weightlifting performance (Table 2). In addition, the contact time, the RSI, and the RSR from the DJs were largely correlated with weightlifting performance (Table 3). Trivial to small correlations were found between the flight time and the jump height from the DJs with weightlifting performance (r ranging from −0.037 to 0.258). In addition, significant positive correlations were found between the snatch and the total with the best individual RSI (Figure 2), while the correlation between the clean and jerk with the RSI was almost significant (r = 0.736, *p* = 0.059, very large). Similarly, significant positive correlations were found between the RSR with the snatch (r = 0.923, *p* = 0.003, nearly perfect), with the total (r = 0.844, *p* = 0.017, very large), and almost with the clean and jerk (r = 0.731, *p* = 0.062, very large).

Isometric peak torque was significantly correlated with the snatch (r = 0.796, *p* = 0.032, very large), almost with the clean and jerk (r = 0.652, *p* = 0.112, large), and nearly with the total (r = 0.744, *p* = 0.054, very large). Small to very large correlations were found between the RTD in all time windows and weightlifting performance (Table 4). 

## 4. Discussion

The purpose of the present study was to investigate the relationship between the RSI during the DJs and the knee extension IPT and RTD with weightlifting performance. The main findings of the study were as follows: (a) the RSI and the contact time calculated from the DJs were significantly correlated with weightlifting performance, (b) individual RSI was significantly positively correlated with the total and the snatch weightlifting performance, and (c) the knee extension IPT and the RTD were moderately to largely correlated with weightlifting performance. These results suggest that the RSI, similar to the CMJ and the SJ, may be a reliable predictor of weightlifting performance, while coaches may consider using the best individual RSI result for predicting weightlifting performance in well-trained male weightlifters. Based also on the current results, a single-joint test, such as the isometric knee extension, may be a moderate predictor of weightlifting performance in well-trained weightlifters. 

As expected, weightlifting performance was significantly correlated with all variables of the CMJ and the SJ. Previous studies have shown that performance in the CMJ and the SJ were significantly correlated with weightlifting performance in both male and female weightlifters [6,7,8,9,10,11,13]. Additionally, a recent meta-analysis showed that performance in the CMJ and the SJ are the best predictors for weightlifting performance [14]. These strong correlations are derived from the biomechanical similarities, and mainly of the lower body triple extension (hip, knee, and ankle extensors), which may be observed in both weightlifting movements and vertical jumping attempts [8,19]. However, whether RSI from the DJ may be correlated with weightlifting performance was unclear. The findings of the present study showed that the contact time, the RSI, and the RSR were the main variables from all DJ heights that were strongly correlated with weightlifting performance. The DJ is a technical demanding vertical jump, in comparison to the CMJ and the SJ, which requires producing a short-time eccentric contraction phase followed by a rapid concentric vertical jump effort [15]. Similarly, during the end of the second pull, in both the snatch and the clean movements, weightlifters drive their bodies under the barbell, emphasizing the strong placement of their lower bodies on the ground. Then, the weightlifters move the barbell overhead (snatch) or on their shoulders (clean), generating a strong whole-body eccentric muscle contraction followed by an abrupt concentric muscle contraction in an attempt to overcome the lifting load and recover in the standing position. Due to these similarities, it might be hypothesized that the RSI and the RSR, as calculated from the DJ, might be strong predictors of weightlifting performance. Therefore, coaches and strength and conditioning professionals may consider using the DJs during training or prior to main competitions for evaluating the athlete’s preparedness before the major competitions. Interestingly, lower correlations were found between the RSI and the clean and jerk, which might be attributed to the presence of the jerk in the movement pattern, masking perhaps a stronger correlation outcome. Although the 1-RM strength in the back squat and the front squat or/and in the power snatch and the power clean may be better predictors for competitive performance [14], coaches may consider using a simple, easily executed and practical field test, like the DJ, which provides an index of explosiveness (RSI, RSR) and is also a good predictor of weightlifting performance. However, these results should be viewed with caution since, according to the authors’ knowledge, this is the first study that investigated the correlation between the RSI and the RSR with weightlifting performance. More research is required to reach certain conclusions.

Comparison between the different DJ heights showed that the flight time and the jumping height were greater for the 40 cm drop height compared to the 20 cm drop height but not when compared with the 30 cm drop height. In addition, no significant difference was observed for the contact time, the RSI, and the RSR between all conditions evaluated. Therefore, both jumping heights from 30 and 40 cm may be optimal to calculate the RSI and the RSR, although an individual drop height should be preferred. These results are in line with a previous study of 45 college athletes from various sports, which showed that stronger athletes can maintain their reactive strength ability during the DJ compared to their weaker counterparts [33]. Weightlifters who participated in the current study were among the strongest athletes in their body mass category in their country. Moreover, the athletes achieved their best RSI and RSR scores from an optimum drop height of approximately 37 cm. A previous study of 17 national-level power and team sport athletes showed that the optimum drop height was 29.4 ± 16.0 cm, although this was calculated from four different drop heights of 20, 40, 60 and 80 cm [27]. Since, in the current study, six out of seven athletes achieved their best RSI and RSR scores from the 40 cm height, it can be speculated that a 40 cm drop height might be a piece of practical and valuable training information for coaches and strength and conditioning professionals for attempting to increase the power performance of their weightlifters. Still, future studies should focus on the investigation of even higher DJ heights in well-trained weightlifters.

A very large correlation was found between the IPT with the snatch and the total, while the clean and jerk was largely positively correlated with the IPT. Similar to the present findings, a study of sixty-seven male weightlifters under the age of 17 showed significant correlations between the isokinetic knee extension strength at 60, 90, and 180 deg/sec and weightlifting performance (r = 0.597, 0.693, and 0.725, respectively) [23]. However, several studies have shown stronger correlations between weightlifting performance and multi-joint isometric tests such as the isometric mid-thigh pull [6,9,13,21] and the isometric leg press [7,10,11]. The knee extension IPT is a single-joint movement involving only the quadriceps muscles. Consequently, coaches may consider using this particular laboratory test with caution for predicting weightlifting future performance. Additionally, large to very large correlations (ranging from 0.581 to 0.766) were found between the knee extension RTD in time windows from 0–60 ms to 0–250 ms with the weightlifting performance. Similar results were found in previous studies using the isometric mid-thigh pull and the isometric leg press in trained (r ranging from 0.62 to 0.76) [13], sub-elite (r ranging from 0.580 to 0.767) [21], well-trained (r ranging from 0.446 to 0.655) [10], and elite weightlifters (females; r ranging from 0.69 to 0.80; males; r ranging from 0.660 to 0.733) [7,9]. Thus, the isometric knee extension RTD may also be a valid test for the evaluation of fast force production and for assessing the preparedness of the neuromuscular system in well-trained weightlifters. However, more studies are required to reach safe, relevant conclusions. 

This study describes the correlation between the RSI, the knee extension IPT, and the RTD with weightlifting performance in well-trained male weightlifters. The small sample size and the different weight mass categories of the athletes may partially limit the generalization of the results. This particular limitation might be partly counterbalanced by the high level of athletic performance and the long-term training experience of the athletes. Still, weightlifting is an explosive sporting event, where muscle fiber types and neural factors may contribute to performance results; neither fiber type composition nor electromyographic activity were examined in the present study, which might have provided a better understanding of the current results. Further studies should examine the role of the DJ from different drop heights (including heights above 40 cm) as well as the knee extension IPT and the RTD in larger groups of weightlifters and in female weightlifters. 

## 5. Conclusions

The contact time, the RSI and the RSR from the DJ may be strong predictors of weightlifting performance in well-trained weightlifters. These results suggest that coaches may regularly include DJs in their training programs in order to increase lower body power and predict the weightlifting performance of their athletes. Moreover, when athletes approach the competition period and the training load-volume is reduced, then coaches may use DJ scores as an index of the muscular explosiveness and readiness of their athletes before main competitions. Although both the 30 and the 40 cm drop heights may effectively be used for all athletes during training, coaches should regularly estimate the optimal individual drop height of each athlete in an attempt to maximize the RSI and the RSR. Additionally, when access to the isometric mid-thigh pull or the isometric leg press performance tests is limited, then the knee extension RTD may be used to predict weightlifting performance and the status of the neuromuscular system of the weightlifter. Therefore, it is suggested that, in line with the CMJ and the SJ power tests, coaches may also effectively use the DJ, the knee extension IPT, and the RTD to predict weightlifting performance in well-trained weightlifters. 

## Figures and Tables

**Table 1:** Weightlifting performance expressed both in absolute (kg) and Sinclair values: countermovement jump, squat jump, lower body isometric peak torque, and rate of torque development results.

Snatch (kg)	100.1 ± 11.2
Clean and Jerk (kg)	124.0 ± 16.0
Total (kg)	224.1 ± 26.1
Snatch (Sinclair)	122.3 ± 12.0
Clean and Jerk (Sinclair)	151.6 ± 18.0
Total (Sinclair)	274.0 ± 28.6
CMJ height (cm)	48.6 ± 9.9
CMJ power (W)	4448.2 ± 659.1
CMJ power/body mass (W/kg)	55.2 ± 6.2
SJ height (cm)	44.3 ± 10.0
SJ power (W)	4278.7 ± 744.0
SJ power per body mass (W/kg)	53.1 ± 7.3
IPT (N·m^−1^)	331.4 ± 51.7
RTD40 (N·m^−1^·s^−1^)	1873.1 ± 444.6
RTD60 (N·m^−1^·s^−1^)	1946.6 ± 439.8
RTD80 (N·m^−1^·s^−1^)	1978.3 ± 397.3
RTD100 (N·m^−1^·s^−1^)	1848.4 ± 331.8
RTD120 (N·m^−1^·s^−1^)	1770.6 ± 342.0
RTD150 (N·m^−1^·s^−1^)	1651.2 ± 314.4
RTD200 (N·m^−1^·s^−1^)	1403.6 ± 255.6
RTD250 (N·m^−1^·s^−1^)	1173.3 ± 215.6

SJ = squat jump, CMJ = countermovement jump, IPT = isometric peak torque, RTD = rate of torque development.

**Table 2:** Correlation coefficients between the squat jump and the countermovement jump with weightlifting performance, expressed with Sinclair formula.

	Squat Jump			Countermovement Jump		
	Jump Height	Power	Power per Body Mass	Jump Height	Power	Power per Body Mass
**Snatch**	0.885 **#	0.753 #	0.890 **#	0.797 *#	0.661 ‡	0.794 *#
**Clean and Jerk**	0.765 *#	0.634 ‡	0.780 *#	0.754 #	0.602 ‡	0.762 *#
**Total**	0.852 *#	0.715 #	0.864 *#	0.808 *#	0.656 ‡	0.813 *#

* *p* < 0.05, ** *p* < 0.01, ‡ large ≤ 0.50–0.69; # very large ≤ 0.70–0.89.

**Table 3:** Correlation coefficients between the drop jump from three different drop heights (20, 30, and 40 cm) with weightlifting performance, expressed with Sinclair formula.

	Drop Jump 20 cm			Drop Jump 30 cm			Drop Jump 40 cm		
	Contact Time	RSI	RSR	Contact Time	RSI	RSR	Contact Time	RSI	RSR
**Snatch**	−0.885 **#	0.866 *#	0.892 **#	−0.899 **#	0.875 **#	0.912 **#	−0.879 **#	0.922 **§	0.920 **#
**Clean and Jerk**	−0.617 ‡	0.704 #	0.684 ‡	−0.599 ‡	0.673 ‡	0.665 ‡	−0.610 ‡	0.710 #	0.689 ‡
**Total**	−0.759 *#	0.806 *#	0.804 *#	−0.754 #	0.790 *#	0.801 *#	−0.753 #	0.833 *#	0.819 *#

RSI = Reactive strength index, RSR = reactive strength ratio, * *p* < 0.05, ** *p* < 0.01; ‡ large ≤ 0.50–0.69; # very large ≤ 0.70–0.89; and § nearly perfect ≥ 0.9.

**Table 4:** Correlation coefficients between the rate of torque development with weightlifting performance, expressed with Sinclair formula.

	RTD 20 ms	RTD 40 ms	RTD 60 ms	RTD 80 ms	RTD 100 ms	RTD 120 ms	RTD 150 ms	RTD 200 ms	RTD 250 ms
**Snatch**	−0.254 ˠ	0.315 †	0.718 #	0.725 #	0.726 #	0.751 #	0.766 *#	0.749 #	0.703 #
**Clean and Jerk**	−0.347 †	0.349 †	0.681 ‡	0.583 ‡	0.660 ‡	0.668 ‡	0.650 ‡	0.651 ‡	0.623 ‡
**Total**	−0.325 †	0.352 †	0.730 #	0.671 ‡	0.720 #	0.735 #	0.730 #	0.724 #	0.687 ‡

IPT = isometric peak torque, RTD = rate of torque development,* *p* < 0.05; ˠ small < 0.10–0.29; † moderate ≤ 0.30–0.49; ‡ large ≤ 0.50–0.69 and # very large ≤ 0.70–0.89.

## Data Availability

The data presented in this study are available upon request from the corresponding author.
